# Evaluation of Nurse-Led Sono-Triage in Identifying the Under-Triage Rate Among Yellow-Triage Patients During Mass Casualty Incidents at a Level 1 Trauma Center in India

**DOI:** 10.7759/cureus.109785

**Published:** 2026-05-27

**Authors:** Geeta Sinha, Sanjeev Bhoi, Tejprakash Sinha, Shakuntala Sundriyal, Shyamala Kondru, Shoukkathali Vazhayil, Radhika Magan

**Affiliations:** 1 Emergency Medicine, All India Institute of Medical Sciences, New Delhi, New Delhi, IND; 2 Accident and Emergency, Tipperary University Hospital, Clonmel, IRL; 3 Biostatistics, All India Institute of Medical Sciences, New Delhi, New Delhi, IND

**Keywords:** mass casualty incident, nurse, sono triage, trauma, triage

## Abstract

Background: Triage is critical during mass casualty incidents (MCIs). Under-triage during MCIs using existing triage tools may lead to errors in making critical treatment decisions. The point-of-care ultrasound has been used during MCIs to diagnose hemoperitoneum, pneumothorax, long bone fractures, cardiac tamponade, and inferior vena cava (IVC), with good sensitivity and specificity.

Objective: We explored the impact of nurse-led sono-triage in identifying the under-triage rate among yellow triage patients and inter-rater agreement among nurse performers during MCIs.

Methods: This was a retrospective observational study of four MCIs during Holi festivals encountered at the emergency department of a level one trauma centre in India. After undergoing training, 10 nurses performed extended focused assessment by sonography in trauma (E-FAST) of yellow triaged patients. The findings were recorded and analyzed.

Results: Sono triage was done in 438 patients. Eight patients were E-FAST positive, and 430 E-FAST negative patients. These eight patients (1.8%) were then triaged as red and categorized as under-triaged. The interrater agreement between the nurse performers and the radiologist was 100%.

Conclusion: Sono-triage was able to estimate the under-triage rate among yellow triage patients during MCIs. The nurses who performed sono-triage had a good inter-rater agreement with the radiologist. Nurses may be utilized as a task-sharing model to do sono-triage during MCIs.

## Introduction

Holi is a festival of color celebrated primarily in India, South Asian countries, and the emigrant ethnic Indian population [1,2]. Health hazards such as dermatological and eye injuries are common due to exposure to the color of Holi. People also get intoxicated with alcohol and opium, leading to a surge of assaults, falls, and road traffic injuries (RTIs). They present to emergency departments (EDs) in large numbers, creating a mass-casualty situation during the Holi festival [3-5].

Mass casualty incidents (MCIs) require quick and reliable triage of large numbers of injured patients [6]. During these MCIs, the ED care providers rely solely on traditional clinical evaluation-based triage, which is relatively time-consuming and often inaccurate, leading to errors.

The authors developed the All India Institute of Medical Sciences (AIIMS) triage protocols and implemented them in 2010. Current triage protocols depend heavily on history and physical examination to determine care priority. An over-triage rate of <35% and an under-triage rate of <5% are considered acceptable during peacetime [7]. Protocol-based triage is critical during MCIs with acceptable under-triage and over-triage rates. However, under-triage during MCIs using existing triage tools may lead to errors in making critical treatment decisions and cause delays in emergency care.

Point-of-care ultrasound (POCUS) is being used as an adjunct to the clinical examination, which improves treatment decision-making for critically ill and injured patients. POCUS is increasingly being used in resource-limited settings. POCUS can decrease medical errors and provide more efficient real-time diagnosis [8].

POCUS is widely used during resuscitation in prehospital and in hospital ED settings. POCUS is commonly utilized for focused assessment by sonography in trauma (FAST) or extended FAST (E-FAST), fracture detection, inferior vena cava (IVC) assessment, and to evaluate cardiac activity during cardiopulmonary resuscitation (CPR) [9].

POCUS has been used during mass casualty, combat, the Armenian earthquake (1988) [10], and the Turkish earthquake (1999) [11] to aid in the diagnosis of hemoperitoneum and pneumothorax; however, it was not used to guide triage decision-making. We hypothesize that POCUS may optimize the existing MCIs triage tool by reducing the under-triage rate [2]. There is a paucity of literature on POCUS-guided triage, especially using FAST or E-FAST during mass casualty events. Stawicki et. al. proposed POCUS-guided triage using the chest, abdomen, vena cava, and extremities in the acute triage (CAVEAT) protocol, but this proposal requires validation [12]. We compared the impact of POCUS-led triage to sonography-led triage (sono-triage) in identifying the under-triage rate among yellow triage patients and compared it to the inter-rater reliability with a radiologist during MCIs at a single level 1 trauma center.

## Materials and methods

This was a single-centre retrospective observational study, which included trauma patients from four MCIs of Holi festivals from 2019 to 2023 visited at the ED of Jai Prakash Narayan Apex Trauma Centre, AIIMS (JPNATC AIIMS), a level 1 trauma centre in New Delhi, India. We included yellow triaged patients as per the AIIMS Trauma Triage Protocol (ATP) of all ages and sexes. Patients requiring immediate resuscitation (red triage) and minor cases (green triage), or patients unwilling to participate in the study, were excluded. This study was approved by the ethics committee.

Yellow-triage patients require monitoring of vital functions and are defined as patients who “have identifiable serious injuries or a suspected mechanism of injury.” These patients require further investigation and continued observation as part of their ongoing care. In the AIIMS triage system, the yellow category corresponds to Emergency Severity Index (ESI) categories 3 or 4 (Table 1).

**Table 1:** Comparison of the AIIMS Triage Protocol (ATP) with other triage tools. AIIMS: All India Institute of Medical Sciences; ESI: Emergency Severity Index; CTAS: Canadian Triage and Acuity Scale; MTS: Manchester Triage Scale; ATS: Australasian Triage Scale

ESI Category	CTAS Category	MTS Category	ATS Category	ATP Category
1, 2	I, II, III	1, 2	1, 2, 3	Red
3, 4	IV	3	3	Yellow
5	V	4, 5	4, 5	Green

Sono-triage was defined as the integration of the AIIMS ATP with the Extended Focused Assessment with Sonography for Trauma (E-FAST) examination.

**Figure 1:**
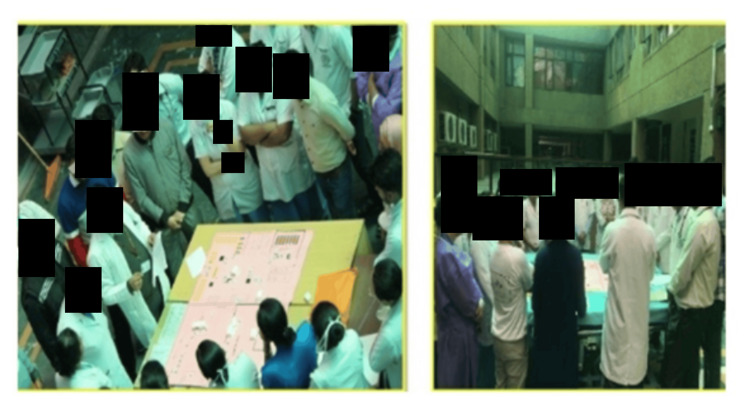
Mass casualty incident preparedness: tabletop drill.

The “Sono-Triage Training” included 10 registered nurses with more than three years of emergency care experience who underwent a four-hour training module consisting of a one-hour didactic lecture and three hours of hands-on skills practice. The training included ultrasound basics, knobology, and the E-FAST examination. After the training, the nurses practiced E-FAST examinations in the trauma bay under the direct guidance and supervision of trained emergency physicians for one week. After this training, each nurse independently performed 20 E-FAST scans (15 normal scans and five abnormal examinations) over a four-week period. All E-FAST scans were recorded and reviewed by senior emergency medicine faculty before patient recruitment. During the Holi festival (which was classified as an MCI), more than 400 patient visits occurred over 24 hours, with the majority occurring during evening and nighttime hours. As per the hospital MCI policy, annual tabletop drills, simulations, debriefings, and other training sessions were provided to responsible team members (Figure 1).

**Figure 2:**
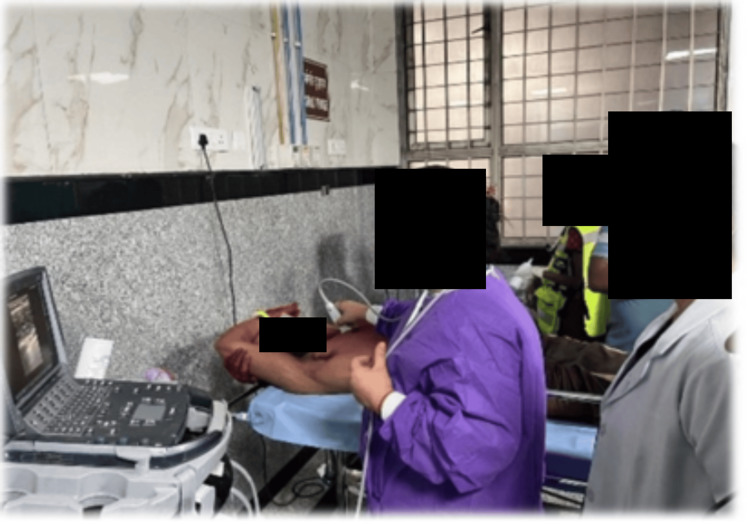
Setup of the sono-triage station.

We created a protocol for sono-triage. Sono-triage stations were created near the triage area during the MCI preparedness scenarios (Figure 2).

Human resources training using the E-FAST protocol for tabletop drills helped clarify the operational details for the execution of the sono-triage in mass casualty management. The sono-triage training and implementation were part of the institution’s routine mass casualty preparedness program.

The study flow included all patients who received care utilizing the defined process-of-care during the MCI. Upon arrival at the ED, all patients were triaged using the ATP. After initial triage, the sono-triage was done by the E-FAST-trained nurses.

Those who were triaged as yellow by the ATP were subsequently sent to the sono-triage station. All patients triaged as yellow underwent an E-FAST scan performed by trained nurses. This was followed by an E-FAST scan performed by a radiologist within 15 minutes of the initial triage. Patients who were E-FAST positive were categorized as red-category patients (ESI categories 1 and 2). Patients with a negative E-FAST finding were categorized as yellow-triage patients (ESI categories 3 and 4).

**Figure 3:**
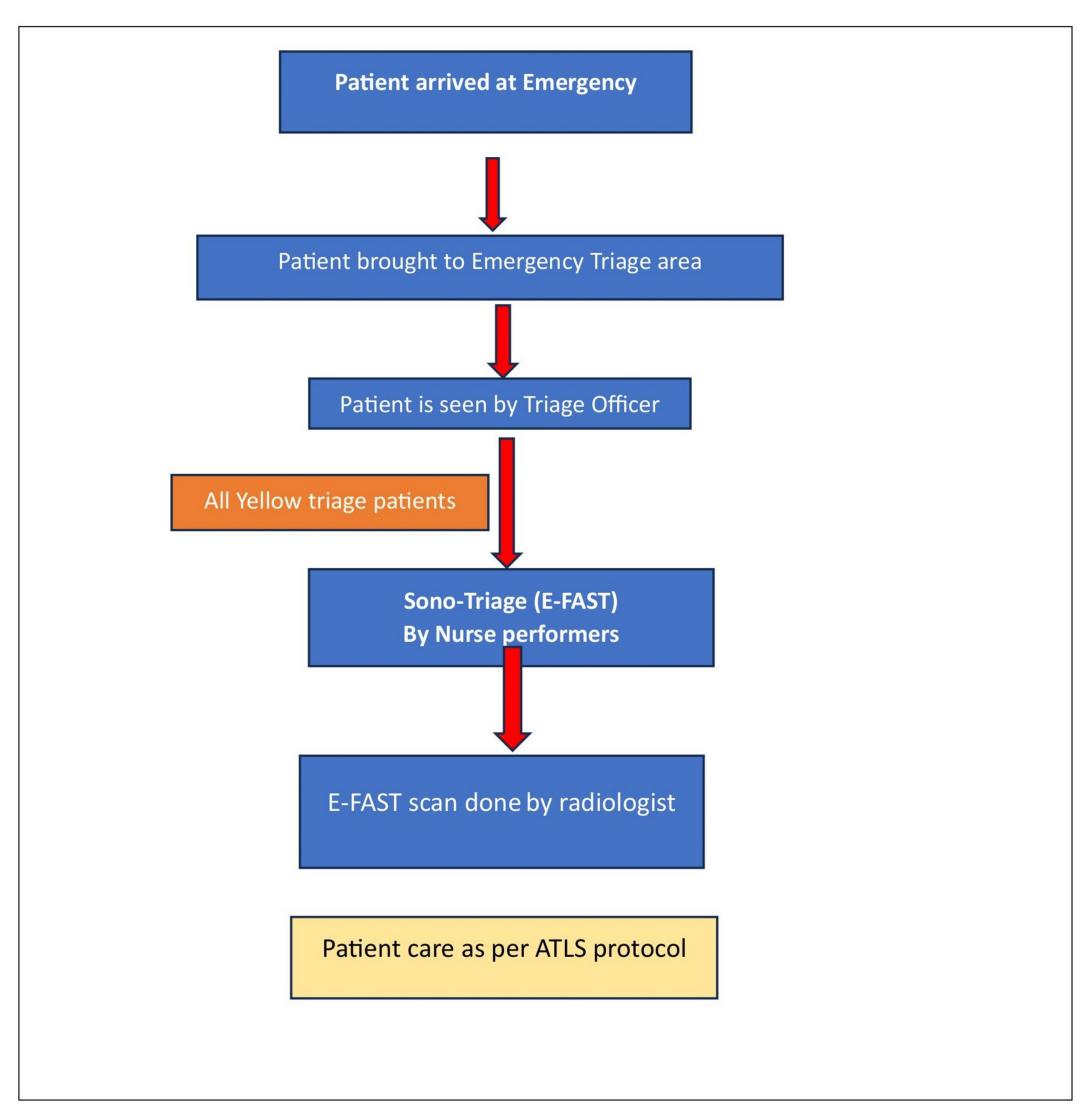
Process of emergency care during mass casualty incidents. E-FAST: extended focused assessment by sonography in trauma; ATLS: Advanced Trauma Life Support

Patients with positive findings on E-FAST were categorized as sono-triage positive. Patients with negative findings on E-FAST were categorized as sono-triage negative (Figure 3).

**Figure 4:**
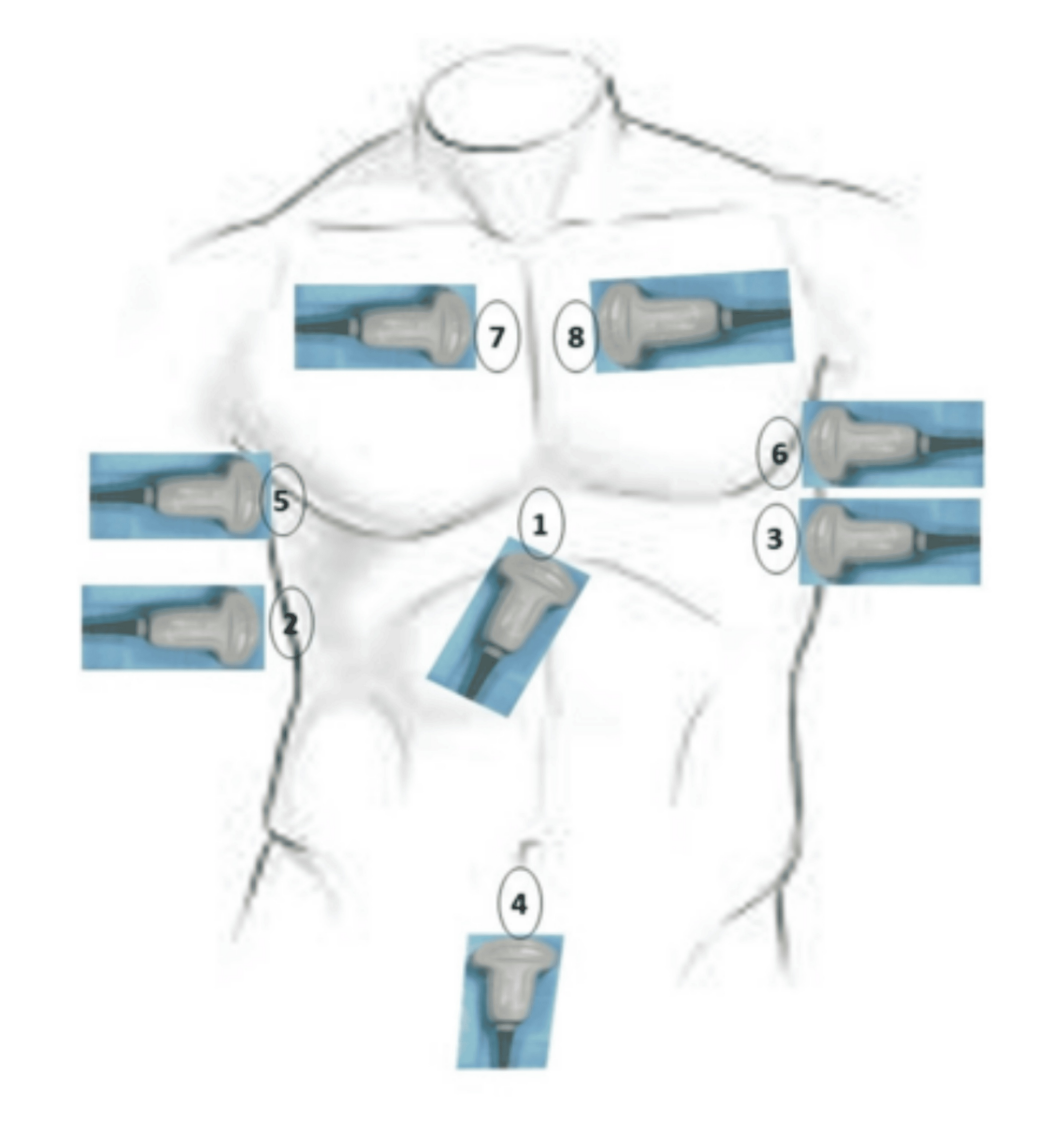
Sono-triage scan windows. 1: subxiphoid; 2: right upper quadrant (RUQ); 3: left upper quadrant (LUQ); 4: pelvic; 5: right basal pleural; 6: left basal pleural; 7: right parasternal; 8: left parasternal Illustration created by the authors using probe pictures.

We adopted a similar method of E-FAST scans done by Giraldo-Restrepo et al. [13]. All abdominal windows were utilized for E-FAST scan as described by the FAST and extended FAST study by Giraldo-Restrepo et al. [13]. Chest scans were done on supine patients with the probe placed perpendicular to the chest wall, with the probe indicator pointing towards the patient’s head (Figure 4).

The anterior chest regions (both sides) included lung zones 1 and 2, corresponding to the second, third, and fourth intercostal spaces (ICS). The axillary regions (both sides) included lung zones 3 and 4, corresponding to the 4th-10th ICS between the anterior and posterior axillary lines. These lung zones were evaluated to rule out hemothorax or pneumothorax using standard lung ultrasound findings. Data were collected using a predesigned proforma attached to the case file for each patient and entered into the medical record. After collecting records of all MCIs, the under-triage rate and inter-rater reliability were calculated.

## Results

A total of 1,609 trauma patients were evaluated in the ED during these four MCIs. Of these, 82.8% were male patients. The common mechanisms of injury (MOI) were assaults in 46% (743) of cases, RTIs in 26.4% (425), and falls in 22.4% (361).

Among them, 44.18% (711) were categorized as yellow following the E-FAST examination and were included in the study. Patients triaged as red (8.7%; 140), green (46.73%; 752), and six patients declared dead on arrival (DOA) were excluded from the study. An additional 273 patients were excluded because sono-triage could not be performed due to technical issues (POCUS machine battery exhaustion) and the unavailability of trained personnel during handover periods (Table 2).

**Table 2:** Patient characteristics, mode of injury, and triage categories. DOA: dead on arrival; RTI: road traffic injury

Variable	Value
Sex distribution (n = 1,609)	
Male	82.8% (1,333)
Female	17.2% (276)
Mode of injury	
RTI	26.4% (425)
Fall	22.4% (361)
Assault	46.0% (743)
Others	5.0% (80)
Triage category	
Red	8.7% (140)
Yellow	44.18% (711)
Green	46.73% (752)
Black (DOA)	0.37% (6)

Sono-triage was performed in 438 patients. Eight patients were E-FAST positive, while 430 patients were E-FAST negative. These eight patients (1.8%) were subsequently triaged as red and categorized as under-triaged. A total of 430 patients (98.2%) were triaged as yellow based on sono-triage findings. Six patients had hemoperitoneum, and two patients had both hemoperitoneum and pericardial effusion. The inter-rater reliability between the trained nurses and the radiologist was 100%. Overall, 74.8% (328) of patients were discharged, while 7.3% were admitted to the hospital (Table 3).

**Table 3:** Results of sono-triage.

Variable	Value
Total patients undergoing sono-triage	27% (438/1,609)
Age group	2 months to 87 years
Pediatric patients	61/438 (13.9%)
Sono-triage negative	430/438 (98.2%)
Sono-triage positive/under-triaged	8/438 (1.8%)
Inter-rater reliability (nurses vs radiologists)	100%; kappa coefficient = 1 (p < 0.0001)
Discharged	328/438 (74.8%)
Admitted	32/438 (7.3%)
Eloped	78/438 (17.8%)

## Discussion

Triage systems need to be quick and reliable, especially during MCIs, where large numbers of victims are transported to the hospital ED [6]. POCUS, in the form of a FAST examination, has been utilized as an adjunct during the initial management of trauma patients. This allows for the rapid detection of hemopericardium and hemoperitoneum. Applications of POCUS have been adopted in disaster settings and acute care for resuscitation, early diagnosis, and procedural guidance [8]. However, its use in guiding triage during MCIs remains limited.

Sarkisian and colleagues first described the use of POCUS during a natural disaster setting in Armenia in 1988 [10]. The study by Dean et al. [14] concluded that hand-carried ultrasound is highly valuable in austere medical settings, particularly following natural disasters, because of its versatility, portability, and noninvasive nature [14]. In another study conducted after the magnitude 7.6 earthquake in Turkey in August 1999, Keven and colleagues examined the prognostic utility of ultrasound in determining the need for dialysis in patients with crush injuries [11].

Estimates of fatalities and casualties from this event vary, but generally reported figures indicate that approximately 17,000 people were killed and another 45,000 were injured [12]. Sztajnkrycer et al., in their study, examined the incidence of positive FAST ultrasonographic findings in trauma patients and found that the use of portable ultrasound technology in disaster settings might identify delayed (yellow-triage) patients with evidence of hemoperitoneum, thereby expediting their transport for definitive care and subsequent management [9].

We used POCUS-guided triage during a unique MCI associated with the Holi festival. Sono-triage identified an under-triage rate of 1.8% among yellow-triaged patients, while 98.2% of patients were triaged appropriately. Sztajnkrycer et al. identified 6.9% (20/286) of patients as yellow-triaged in a similar study using POCUS-guided triage. Six patients underwent operative management within 24 hours. In that study, one out of nine patients identified through sono-triage underwent operative management within 24 hours. They utilized emergency physicians and surgeons to perform POCUS-guided triage in disaster settings. By using FAST examinations, emergency physicians and surgeons can be better utilized for resuscitation and operative management rather than triage [9]. We utilized trained nurses to perform POCUS-guided triage. This task-sharing model was developed so that doctors could lead the resuscitation team. The study compared the inter-rater agreement between nurses and the radiologist. The inter-rater agreement was 100% (kappa coefficient = 1; p < 0.0001). Previous studies reported that nurse-performed FAST achieved similar accuracy to doctor-performed FAST [15]. This reflects the quality of training provided to the nurses before recruitment into the study. POCUS improved diagnosis and assisted clinical decision-making regarding whether patients required referral to higher levels of care, which is crucial in resource-limited settings and encourages the use of health services. The combination of accurate diagnosis, effective treatment, and appropriate referrals leads to improved patient outcomes [16].

Ten registered nurses with more than three years of emergency care experience participated in a structured four-hour sono-triage training module, which included a one-hour didactic session and three hours of hands-on practical training, as part of the institution's regular disaster preparedness and mass casualty management training. The curriculum covered E-FAST assessment methods, knobology, and the basics of POCUS. After the initial training, nurses spent a week doing supervised E-FAST assessments in the trauma bay under the supervision of qualified emergency physicians. After that, over the course of four weeks, each nurse independently finished 20 supervised E-FAST scans (15 normal and five abnormal examinations). Before being used independently in clinical settings, all scans were examined and verified by senior emergency faculty. E-FAST exams were then carried out by qualified nurses as part of the predetermined sono-triage workflow. The institution's standard role-based mass casualty preparedness program, which also comprised yearly tabletop exercises, simulations, and debriefing sessions for emergency workers, included this sono-triage training, which was not created especially for the current study.

AIIMS sono-triage and the ATP were used in this study. We used this protocol because its learning curve is quick, and execution was operationally easier compared with the CAVEAT protocol described by Stawicki et al. The CAVEAT protocol suggests abdominal, thoracic, vena cava, and extremity assessment for long-bone fractures using POCUS in a two-tiered system [11]. However, this protocol has not been validated in any study (Table 4).

**Table 4:** Comparison of AIIMS ST with CAVEAT protocol and FAST as an adjunct to triage. AIIMS: All India Institute of Medical Sciences; ST: sono-triage; CAVEAT: chest, abdomen, vena cava, and extremities in acute triage; FAST: focused assessment with sonography for trauma; START: simple triage and rapid treatment; MCI: mass casualty incident

AIIMS ST	CAVEAT Protocol	FAST as an Adjunct to Triage
Less time-consuming	Time-consuming	Time-consuming
No sonologist required	Sonologist required	Emergency physicians and surgeons required
AIIMS sono-triage protocol is feasible for use in the emergency department	Feasibility of the CAVEAT protocol in the emergency department remains to be established	START has not been validated to support the proposed benefits to victims
Time required for execution is shorter, and the learning curve for nurses is less	Time required for execution may be longer, and the learning curve for nurses may be greater	Patients who might otherwise have been categorized as green were potentially classified as yellow in this study
Does not have multiple components	Has multiple components	-
AIIMS ST protocol detects most life-threatening conditions	The CAVEAT protocol does not detect most intracranial, pulmonary, retroperitoneal, or pelvic injuries	Does not assess fracture detection, pneumothorax, or hemothorax, which are life-threatening conditions
AIIMS ST protocol is yet to be validated	CAVEAT protocol is yet to be validated	The study does not support the routine use of FAST ultrasound as a secondary triage tool during MCIs

A total of 1,609 patients visited the ED during four MCI events associated with the Holi festival. MCIs are usually caused by terror attacks, earthquakes, major road traffic crashes, and mass gatherings, leading predominantly to traumatic injuries. During Holi, people often become inebriated with alcohol and engage in assaults, resulting in an MCI. Ocular toxicity, chemical injuries, and dermatological issues related to the colors used during Holi represent unique patient characteristics [17].

Limitations

There were several limitations. This was a single-center retrospective study of facility-based triage involving only yellow-triaged patients. Although all eligible patients from specific mass casualty situations during a predetermined period were included in the study, no formal sample size calculation was performed. This may reduce the statistical power and generalizability of the findings.

The protocol included only E-FAST and did not explore the role of airway assessment, optic nerve sheath diameter measurement, or fracture identification. Intra-rater agreement among the nurses and the time required to perform sono-triage were not assessed. We suggest prospective multicenter studies on ultrasound-guided primary and secondary triage irrespective of the initial triage category. A comprehensive and feasible algorithm needs to be developed. Further research is also needed regarding its use in field triage settings with handheld ultrasound devices among nonphysician providers such as paramedics and nurses.

## Conclusions

Sono-triage was able to estimate the under-triage rate among yellow-triage patients during MCIs. Sono-triage performed by nurses demonstrated good inter-rater agreement with the radiologist. Nurses can be utilized within a task-sharing model to perform sono-triage during MCIs for rapid assessment and improved patient prioritization. Nurse-led sono-triage may improve triage accuracy.

## References

[1] Ghosh SK, Bandyopadhyay D, Verma SB (2012). Cultural practice and dermatology: the "Holi" dermatoses. Int J Dermatol.

[2] (2026). Holi around the world. https://www.holifestival.org/holi-around-the-world.html.

[3] Belhadjali H, Ghannouchi N, Amri Ch, Youssef M, Amri M, Zili J (2008). Contact dermatitis to henna used as a hair dye. Contact Dermatitis.

[4] Majoie IM, Bruynzeel DP (1996). Occupational immediate-type hypersensitivity to henna in a hairdresser. Am J Contact Dermat.

[5] Ventura MT, Di Leo E, Buquicchio R, Foti C, Arsieni A (2007). Is black henna responsible for asthma and cross reactivity with latex?. J Eur Acad Dermatol Venereol.

[6] Burkle FM Jr (2002). Mass casualty management of a large-scale bioterrorist event: an epidemiological approach that shapes triage decisions. Emerg Med Clin North Am.

[7] (2026). Resources for optimal care of the injured patient. https://www.facs.org/quality-programs/trauma/quality/verification-review-and-consultation-program/standards/.

[8] Moore CL, Copel JA (2011). Point-of-care ultrasonography. N Engl J Med.

[9] Sztajnkrycer MD, Baez AA, Luke A (2006). FAST ultrasound as an adjunct to triage using the START mass casualty triage system: a preliminary descriptive system. Prehosp Emerg Care.

[10] Sarkisian AE, Khondkarian RA, Amirbekian NM, Bagdasarian NB, Khojayan RL, Oganesian YT (1991). Sonographic screening of mass casualties for abdominal and renal injuries following the 1988 Armenian earthquake. J Trauma.

[11] Auth -Marza V: (2026). On the death toll of the 1999 Izmit (Turkey) major earthquake. Potsdam: European Seismological Commission.

[12] Stawicki SP, Howard JM, Pryor JP, Bahner DP, Whitmill ML, Dean AJ (2010). Portable ultrasonography in mass casualty incidents: the CAVEAT examination. World J Orthop.

[13] Giraldo-Restrepo JA, Serna-Jiménez TJ (2015). The FAST and extended FAST examinations. Colomb J Anesthesiol.

[14] Dean AJ, Ku BS, Zeserson EM (2007). The utility of handheld ultrasound in an austere medical setting in Guatemala after a natural disaster. Am J Disaster Med.

[15] Varndell W, Topacio M, Hagness C, Lemon H, Tracy D (2018). Nurse-performed focused ultrasound in the emergency department: a systematic review. Australas Emerg Care.

[16] Abrokwa SK, Ruby LC, Heuvelings CC, Bélard S (2022). Task shifting for point of care ultrasound in primary healthcare in low- and middle-income countries-a systematic review. EClinicalMedicine.

[17] Velpandian T, Saha K, Ravi AK, Kumari SS, Biswas NR, Ghose S (2007). Ocular hazards of the colors used during the festival-of-colors (Holi) in India - malachite green toxicity. J Hazard Mater.

